# Editorial: Coronavirus Disease (COVID-19): Pathophysiology, Epidemiology, Clinical Management and Public Health Response

**DOI:** 10.3389/fpubh.2021.807159

**Published:** 2021-11-30

**Authors:** Denise L. Doolan, Zisis Kozlakidis, Zhongheng Zhang, Slobodan Paessler, Longxiang Su, Yasuko Tsunetsugu Yokota, Tatsuo Shioda, Alexander Rodriguez-Palacios, Ata Murat Kaynar, Rukhsana Ahmed, Abdallah Samy, Hannah Bradby, Alexis M. Kalergis, Mohan Jyoti Dutta, Michael Kogut, Shen-Ying Zhang

**Affiliations:** ^1^Australian Institute of Tropical Health and Medicine, James Cook University, Townsville, QLD, Australia; ^2^International Agency for Research on Cancer, World Health Organization, Lyon, France

**Keywords:** coronavirus—COVID-19, first wave, pathophysiology, epidemiology, clinical management, public health response

 During a pandemic, there are multiple concurrent clinical and scientific priorities, including the need to understand the pathophysiology of the disease, the different modes of transmission, how patient care can be optimized, as well as the need to develop mathematical models that can now cast and forecast the progression of infections within given populations and/or geographical regions.

When the current SARS-CoV2 pandemic was declared a Public Health Emergency of International Concern by the World Health Organization, a formal declaration of its gravity, it became evident that there was an acute need to understand all of the above aspects. In doing so, by 11th February 2020, a special topic, entitled “Coronavirus Disease (COVID-19): Pathophysiology, Epidemiology, Clinical Management and Public Health Response,” was initiated with a dedicated team of handling editors to facilitate the timely peer-review and publication of relevant manuscripts (1). Frontiers, as the publisher of this special topic, took the bold step of waiving any article processing charges so that financial constraints would not be a barrier to communicating crucial information about the pandemic to a broad audience. Furthermore, this was the most extensive special topic to date in the Frontiers portfolio, in terms of the numbers of participating *Frontiers* journals, disciplines, and sections. This reflected the acute need for the scientific community to understand the many aspects of the pandemic.

This special Research Topic captured the entire first wave in the northern hemisphere, from February to May 2020, and the intensity of the associated editorial work is evident by the reported numbers. Within 4 months, 194 abstracts were received; in total 851 manuscripts were submitted, of which 453 were rejected while 398 were published. From the scientific community perspective, by June 2020 the special topic achieved over 2 million views, by December 2020 over 4 million views, and by August 2021 over 8 million views. As an example of the breadth of subjects covered, manuscripts included the attempt by Larsen et al. to model the onset of symptoms of COVID-19; the observed gender differences on COVID-19 patients' severity and mortality by Jin et al., the correlation between poverty levels and rates of COVID-19 incidence and death in the United States by Finch and Finch, as well as the careful review of the cytokine storm in COVID-19 (Tang et al.).

However, we must emphasize a note of caution, as some of the published manuscripts could have competing analytical approaches, idiosyncrasies in the reporting of their data, or differences in interpretation of some observations across the many different settings (Struelens et al.). Similarly, it must be recognized that external validation of proposed solutions may not have been possible within the time-frame of the first wave of the pandemic, balanced with the need to rapidly communicate information that would facilitate an effective solution to end the pandemic.

Notwithstanding the above, this special topic reflects a substantial investment by the research community, the supporting editorial team, and the publishing house to facilitate scientific discovery in times of crisis. Thus, even though the special topic lasted for a short window of time, compared to the pandemic's overall duration, its impact should provide us with a renewed optimism that science can continue playing a prominent role in addressing the significant health challenges of our times.

## Author Contributions

All authors listed have made a substantial, direct, and intellectual contribution to the work and approved it for publication.

## Author Disclaimer

Where authors are identified as personnel of the International Agency for Research on Cancer/WHO, the authors alone are responsible for the views expressed in this article and they do not necessarily represent the decisions, policy or views of the International Agency for Research on Cancer/WHO.

## Conflict of Interest

The authors declare that the research was conducted in the absence of any commercial or financial relationships that could be construed as a potential conflict of interest.

## Publisher's Note

All claims expressed in this article are solely those of the authors and do not necessarily represent those of their affiliated organizations, or those of the publisher, the editors and the reviewers. Any product that may be evaluated in this article, or claim that may be made by its manufacturer, is not guaranteed or endorsed by the publisher.
